# High burden of tuberculosis infection and disease among people receiving medication-assisted treatment for substance use disorder in Tanzania

**DOI:** 10.1371/journal.pone.0250038

**Published:** 2021-04-29

**Authors:** Lilian Tina Minja, Jerry Hella, Jessie Mbwambo, Cassian Nyandindi, Ubuguyu Said Omary, Francis Levira, Stellah Mpagama, Meshack Shimwela, James Okuma, Sebastien Gagneux, R. Douglas Bruce, Klaus Reither

**Affiliations:** 1 Ifakara Health Institute, Dar es Salaam, Tanzania; 2 Swiss Tropical and Public Health Institute, Basel, Switzerland; 3 University of Basel, Basel, Switzerland; 4 Muhimbili National Hospital, Dar es Salaam, Tanzania; 5 Muhimbili University of Health and Allied Sciences, Dar es Salaam, Tanzania; 6 Drug Control and Enforcement Authority, Dar es Salaam, Tanzania; 7 Ministry of Health, Community Development, Gender, Elderly and Children, Dodoma, Tanzania; 8 Kibong’oto Infectious Disease Hospital, Moshi, Tanzania; 9 Temeke Regional Referral Hospital, Dar es Salaam, Tanzania; 10 Boston University School of Medicine, Boston, Massachusetts, United States of America; The University of Georgia, UNITED STATES

## Abstract

**Objective:**

To determine the prevalence of tuberculosis (TB) disease and infection as well as incident TB disease among people who use drugs (PWUD) attending Medication Assisted Treatment (MAT) clinics in Dar-es-Salaam, Tanzania.

**Methods:**

In this prospective cohort study, a total of 901 consenting participants were enrolled from November 2016 to February 2017 and a structured questionnaire administered to them through the open data kit application on android tablets. Twenty-two months later, we revisited the MAT clinics and reviewed 823 of the 901 enrolled participant’s medical records in search for documentation on TB disease diagnosis and treatment. Medical records reviewed included those of participants whom at enrolment were asymptomatic, not on TB disease treatment, not on TB preventive therapy and those who had a documented tuberculin skin test (TST) result.

**Results:**

Of the 823 medical records reviewed 22 months after enrolment, 42 had documentation of being diagnosed with TB disease and initiated on TB treatment. This is equivalent to a TB disease incidence rate of 2,925.2 patients per 100,000 person years with a total follow up time of 1,440 person-years. At enrolment the prevalence of TB disease and TB infection was 2.6% and 54% respectively and the HIV prevalence was 44% and 16% among females and males respectively.

**Conclusion:**

PWUD attending MAT clinics bear an extremely high burden of TB and HIV and are known to have driven TB epidemics in a number of countries. Our reported TB disease incidence is 12 times that of the general Tanzanian incidence of 237 per 100,000 further emphasizing that this group should be prioritized for TB screening, testing and treatment. Gender specific approaches should also be developed as female PWUDs are markedly more affected with HIV and TB disease than male PWUDs.

## Introduction

Tuberculosis (TB) is the leading cause of death from a single infectious agent worldwide. In 2019, 10 million people developed TB and 1.408 million lives were lost to TB. In Tanzania, an estimated TB incidence rate of 237 cases per 100,000 population was reported in 2019 with 54% (74,067) of the cases notified, leaving 62,933 TB cases undiagnosed or unreported [1].

Tanzania is a transit country for illicit drugs, particularly heroin, with a growing domestic user population. It is estimated that about 250,000 drug users [2], of which 25,000 to 50,000 are identified as injecting drugs live in Tanzania [3]. Overall, people who *use* drugs (PWUD) are a significant, but underserved group with high risk of TB infection and disease. Risk factors predisposing PWUDs to develop TB disease include HIV co-infection, incarceration, inadequate living conditions, frequent homelessness, poor nutrition, alcohol use disorders, smoking and treatment barriers [4–10]. Furthermore, a practice of inhaling and exhaling or blowing smoke from crack or marijuana directly into another person’s mouth known as “shot gunning” has been reported as a mode of TB transmission [11]. lllicit drugs increase susceptibility to various infections including mycobacterium due to impairment of the cell mediated immune response [10, 12, 13]. Moreover, illicit drug effects often mask symptoms of TB disease leading to a delayed or missed diagnosis [8].

Although substance abuse disorder and TB disease are recognised as a growing public health problem, reports on the burden of TB disease and infection among PWUD in Tanzania are still scarce. One study conducted in 2014 among people who *inject* drugs (PWID) in a Medication Assisted Treatment (MAT) clinic at Muhimbili National Hospital, Dar-es-Salaam, reported a prevalence of active pulmonary TB at 4% [14]. However, there is lack of reports on the prevalence of TB infection among PWUDs, not only for Tanzania but also for sub-Saharan Africa in general. Cohort studies conducted in countries out of Africa, report the prevalence of LTBI among PWUDs to be higher than in the general population ranging from 13 to 59% [8]. Yet, this may not be a true representation in Tanzania due to the different geographic TB disease prevalence.

The presented study investigates the prevalence and incidence of TB disease as well as prevalence of TB infection with associated risk factors among PWUD (this includes both people having used injectable or non-injectable drugs) receiving methadone at MAT clinics in Dar-es-Salaam.

## Materials and methods

### Study site and study participants

This prospective study was conducted at all three MAT clinics that were present in Tanzania at the time of study enrolment, located in Dar-es-Salaam region at the Muhimbili, Mwananyamala and Temeke hospitals. MAT programs were started in 2011 by the government of Tanzania in partnership with Pangaea Global AIDS Foundation with funding from the US Centre for Disease Control through the President’s Emergency [15]. Currently, there are eleven MAT clinics in the country of which ten are in Tanzania mainland and one in Zanzibar attending to a total of about ten thousand (10,000) clients. The MAT clinics in Tanzania mainland are located in different regions namely three in Dar es Salaam, two in Pwani (Bagamoyo and Kibaha) and one each in Tanga, Arusha, Mwanza, Dodoma and Mbeya regions. The clinics provide daily methadone free of charge as a directly observed therapy to PWUD.

Dar-es-Salaam is the most populous region in Tanzania with an estimated 5.3million inhabitants [16] and accounted for 21% of the annual notified TB cases in 2017 [17]. At the time of our study Dar es Salaam was divided into three municipals with each having one MAT clinic i.e. Muhimbili, Mwananyamala and Temeke MAT clinics located within the Ilala, Kinondoni and Temeke municipals respectively. Illicit drugs used in the region include heroin, cocaine, cannabis with modes of administration including smoking, sniffing, inhaling and injecting [18]

We obtained a list of all 3000 clients attending the three MAT clinics and entered it into a computer random number generator. Based on the sample size calculation, a list of 906 clients was generated for enrolment into the study. The inclusion criteria were: Adults (18 years or older), who were known to have used illicit drugs on methadone maintenance treatment and voluntarily consented to participate in the study. The exclusion criterion was unwillingness to participate in the study.

### Sample size

The sample size was calculated by applying finite population correction factor [18]. Due to lack of studies of TB infection among PWUDs in Tanzania, we had to use the prevalence of LTBI as reported in studies out of Africa which ranges between 13 and 59% [8]. In order to be able to determine 35% prevalence of TB infection with 3.5% precision and 95% CI, a sample size of 714 was required. To account for 20% non-response and after excluding 2 participants aged less than 18 years and 3 participants who were not willing to participate, 901 participants were enrolled into the study.

### Data collection and interpretation

#### Enrolment

From November 2016 to February 2017, data was collected through a pretested structured questionnaire developed by the investigators and administered by trained clinicians to each consenting participant. Information collected included socio-demographic indicators including sex, age, gender, residence, occupation and education level. TB risk factors assessed included: i) illicit drug use defined as ever used heroin and or cocaine with or without marijuana ii) method of administering illicit drug defined as ever injected or never injected, without differentiating between previous or current use, iii) history of incarceration or jail detention in the past two years, iv) history of smoking cigarettes or drinking alcohol, v) HIV status (medical records), and vi) self-report and medical records of prior or current TB disease.

A participant was said to have TB disease if they had undergone investigations for TB and found to have active TB and initiated on TB treatment. TB treatment was defined as use of four or more TB medications for not less than 6 months divided into an intensive and continuation phase. TB treatment was used as an indicator of TB disease. Prior or current use of TB preventive therapy was also assessed. TB preventive therapy was defined as a six months course of isoniazid preventive therapy after ruling out TB disease which is provided in accordance to the Tanzania National TB guidelines.

The WHO TB screening questionnaire which includes the following symptoms: current cough, fever, excessive night sweats and weight loss was used to screen for TB disease [19]. Participants responding yes to any of the WHO TB screening questions were classified as presumptive TB and were referred for further investigations to rule out TB disease as per National guidelines. Clinical examination, BCG (Bacille Calmette-Guerin) scar examination on the arm as evidence of previous BCG vaccination, and administration of tuberculin skin test (TST) was done. We performed the TST using tuberculin purified protein derivative (PPD) RT23 (2 tuberculin units, Statens Serum Institut, Copenhagen, Denmark). The test was administered by trained clinicians on the volar surface of the participants left arm (unless contraindicated) using a disposable 1.0ml graduated syringe with instructions on proper care of the site. The transverse induration (if present) was measured 48 to 72 hours post TST administration, read and documented by two independent clinicians using a flexible transparent ruler. TST was considered positive when an induration of ≥ 5mm or ≥ 10mm for HIV positives and negatives respectively was observed in asymptomatic participants [21]. TB infection was defined as a positive TST in an asymptomatic participant, which could be latent or subclinical TB [20]. Anthropometric data included weight and height which was used to determine participants’ body mass index according to WHO classification [21].

Data was captured using the open data kit application (www.opendatakit.org) on Android tablets, with real time error, range and consistency checks. Data management was done using an *e*Management tool “odk planner” [22]. Participants with symptoms or signs suggestive of active TB disease were referred to the National Tuberculosis clinics for further management.

#### Follow up

Review of medical records of enrolled participants after excluding those on TB treatment or TB preventive therapy, presumptive TB cases and those missing TST results at enrolment was done from February to March 2019. Information collected included TB treatment initiated between March 2017 to Jan 2019 and TB diagnostic method (sputum–when TB treatment was initiated based on clinical symptoms and a sputum sample either positive on Xpert MTB/RIF or Acid Fast Bacilli positive on Ziehl Neelsen stain, radiological–when TB treatment was initiated based on clinical symptoms and chest x-ray findings suggestive of TB with negative or no sputum results, clinical–when TB treatment was solely initiated on clinical grounds with negative or no sputum results or chest x-ray findings). History of TB treatment was used as an indicator of TB disease. Time of follow up was censored to the participant’s last visit at the MAT clinic. At the MAT clinics, PWUD were classified as, dead (death of the client), defaulter (missed 28 or more consecutive days of methadone dose), expelled (discharged from methadone treatment for the safety or wellbeing of the client, other clients or the staff members when they violate conditions under which they signed an agreement to participate voluntarily on the MAT services), successful completion (successfully weaned off drugs and methadone), active (daily attendance to MAT clinics for methadone or not meeting the definition of defaulter or expelled or dead or successful completion).

### Data analysis

Baseline socio-demographic and clinical characteristics were presented as percentages and median age in years with interquartile range. We used odds ratios (OR) and 95% confidence intervals (CI) from logistic regression model to estimate predictors of LTBI. We calculated incident rates by taking the number of incident TB diagnosed divided by the person time followed up. Rates were presented per 100,000 person years of follow up, and 95% confidence intervals were calculated using Breslow method of ties. Our primary outcomes were prevalence (LTBI) and incidence (TB). Cox proportional hazard was used to evaluate association between baseline/enrolment characteristics and time to incident TB after enrolment. Patients who did not develop incident TB were censored when they met the definition of defaulter, successful completion, expulsion from MAT clinics, death or last visit date of 30^th^ January 2019. Data was analysed using STATA version 14 (Stata Corp., Texas, USA).

### Ethical considerations

The study was reviewed and approved by the Ethikkommission Nordwest-und Zentralschweiz (EKNZ), Ifakara Health Institute Institutional Review Board (IHI IRB) and the Tanzania National Institute for Medical Research (NIMR). Clearance to conduct the study was provided by NIMR. Permission to conduct the research in the hospitals was obtained from the Regional Medical Officer as well as from the respective hospitals and MAT clinics.

Written informed consent was obtained from each participant prior to any study procedure. In case of illiteracy, study information was given in the presence of an impartial, literate witness, who read the information sheet to the participant or witnessed the complete reading of the information sheet to the participant. The participant thereafter gave consent by thumb printing the ICF. The witness stated that free, informed consent was given by his/her signature on the ICF.

All participants were assigned a study identification number that was used on all the participant’s records. Names or any personal identifiers were not used in order to maintain confidentiality. The investigator ensured that study logs showing identification numbers, participant names and date of births were kept in lockable fireproof cabinets to which only authorised personnel had access.

## Results

### Participant’s characteristics

**Fig 1:**
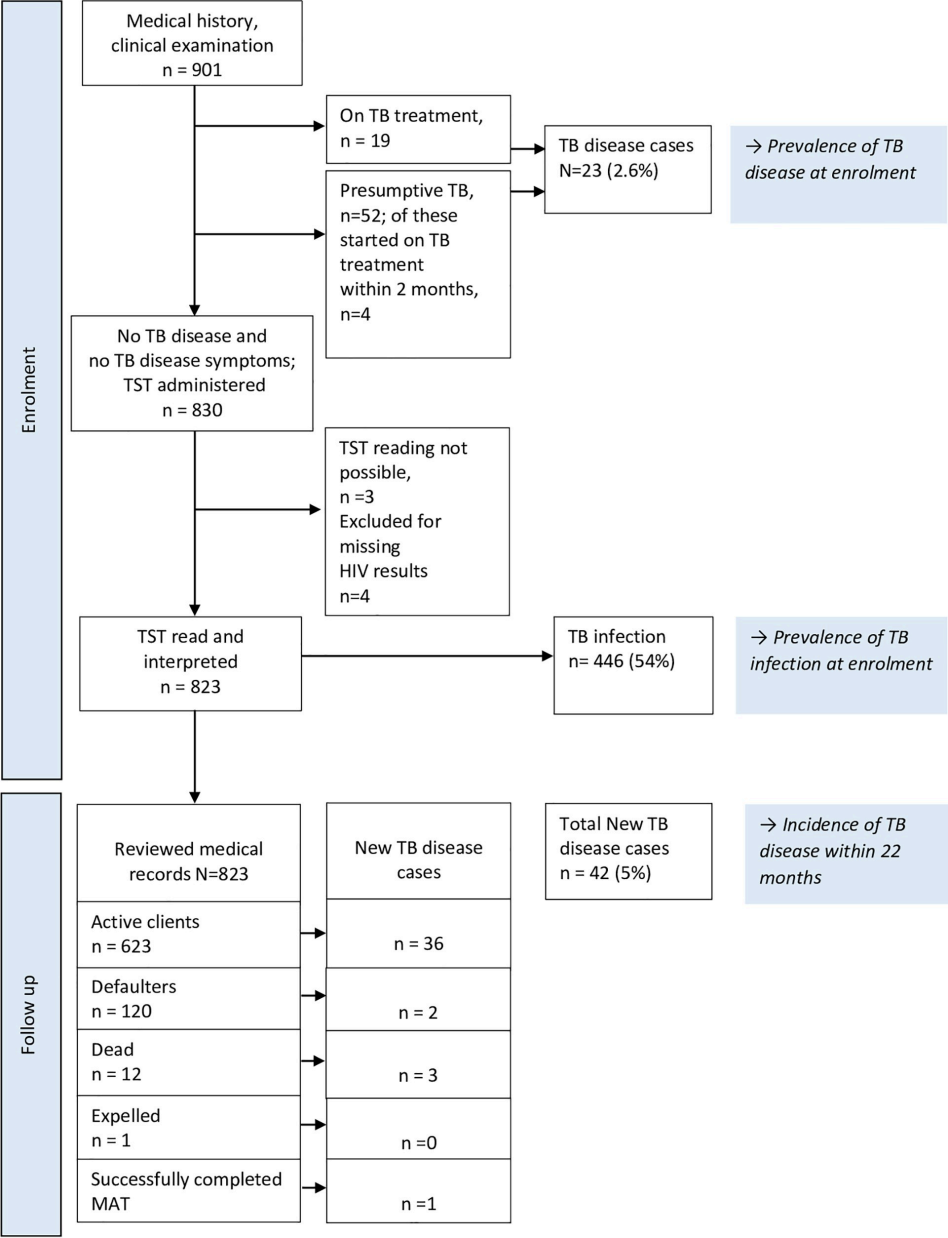
Patient flow diagram.

A total of 901 participants were included into the study. The baseline characteristics and flow are shown in Table 1 and Fig 1 respectively. At enrolment, the median age of study participants was 36 years (IQR 32–41), 805/901 (89.4%) were men, 627/901 (69.6%) had primary school education or less, unemployment was reported by 653/901 (72.5%). Previous history of TB disease was reported by 156/901 (17.3%) and of these 27/156 (17.3%) had two or more prior episodes. Incarceration or jail detention within the previous two years was reported by 524/901 (58.2%). Of the 897/901 (99.6%) with a reported HIV status, 169/897 (18.8%) were HIV positive with prevalence of HIV among females and males at 43.8% and 15.8% respectively. More than half of the participants (53.5%; 482/901) were PWID, and of these 23.0% were HIV positive. History of smoking cigarettes, marijuana and alcohol use was reported by 573/901 (63.6%), 296/901 (32.9%) and 199/901 (22.1%) respectively, without differentiating prior or current use. A BCG scar was observed in 847/901 (94.0%) of the participants. None of the clients had ever received isoniazid preventive therapy (IPT).

**Table 1:** Baseline socio-demographic and clinical characteristics among people receiving medication-assisted treatment for substance use disorder in Dar es Salaam, Tanzania (N = 901).

Characteristics	All participants, N = 901^a^
Age (years), median (IQR)	36 (32–41)
Age categories (years), n(%)	
<30	123 (13.7%)
30–39	500 (55.5%)
40–49	244 (27.1%)
≥50	34 (3.8%)
Sex, Males, n(%)	805 (89.4%)
HIV status, n(%)^b^	
Positive	169 (18.8%)
Negative	728 (81.2%)
Missing	4 (0.4%)
TB status, n(%)	
On TB treatment	19 (2.1%)
Symptoms suggestive of TB	52 (5.9%)
No symptoms suggestive of TB	830 (94.1%)
Previous history of TB, n(%)	156 (17.3%)
Prior TB episodes, n = 156, n(%)	
One	129 (82.7%)
Two or more	27 (17.3%)
Body Mass Index categories, n(%)	
Underweight	135 (15.0%)
Normal weight	644 (71.4%)
Overweight	106 (11.8%)
Obese	16 (1.8%)
Education level, n(%)	
Primary or less	627 (69.6%)
Secondary or more	274 (30.4%)
Occupation, n(%)	
Unemployed	653 (72.5%)
Employed	248 (27.5%)
Residence, Homeless, n(%)	147 (16.3%)
Live with substance abuser, n(%)	149 (16.5%)
Ever injected drugs, n(%)	482 (53.5%)
Alcohol use, n(%)	199 (22.1%)
Smoking cigarettes, n(%)	573 (63.6%)
Smoking marijuana, n(%)	296 (32.9%)
Incarceration or jail detention (last 2 years), n(%)	524 (58.2%)
Health facility, n(%)	
Muhimbili	235 (26.1%)
Mwananyamala	335 (37.2%)
Temeke	331 (36.7%)

^a^Results are number and %. For age in years we also report on median and IQR (Interquartile range).

^b^Results are number and % of those with non-missing HIV data; missing data category is the number and % of missing HIV data.

### Prevalence of TB disease

At enrolment, 19/901 (2.1%) participants were on TB treatment. Of the remaining participants not on TB treatment, 52/882 (5.9%) had symptoms suggestive of TB disease and were classified as presumptive TB. Presumptive TB cases were referred for further investigations and out of these an additional 4/901 (0.4%) were diagnosed to have TB disease and started on TB treatment (Fig 1). Accordingly, the overall TB disease prevalence at enrolment was 23/901 (2.6%) translating to 2,553 per 100,000 people.

### Prevalence of TB infection

**Fig 2:**
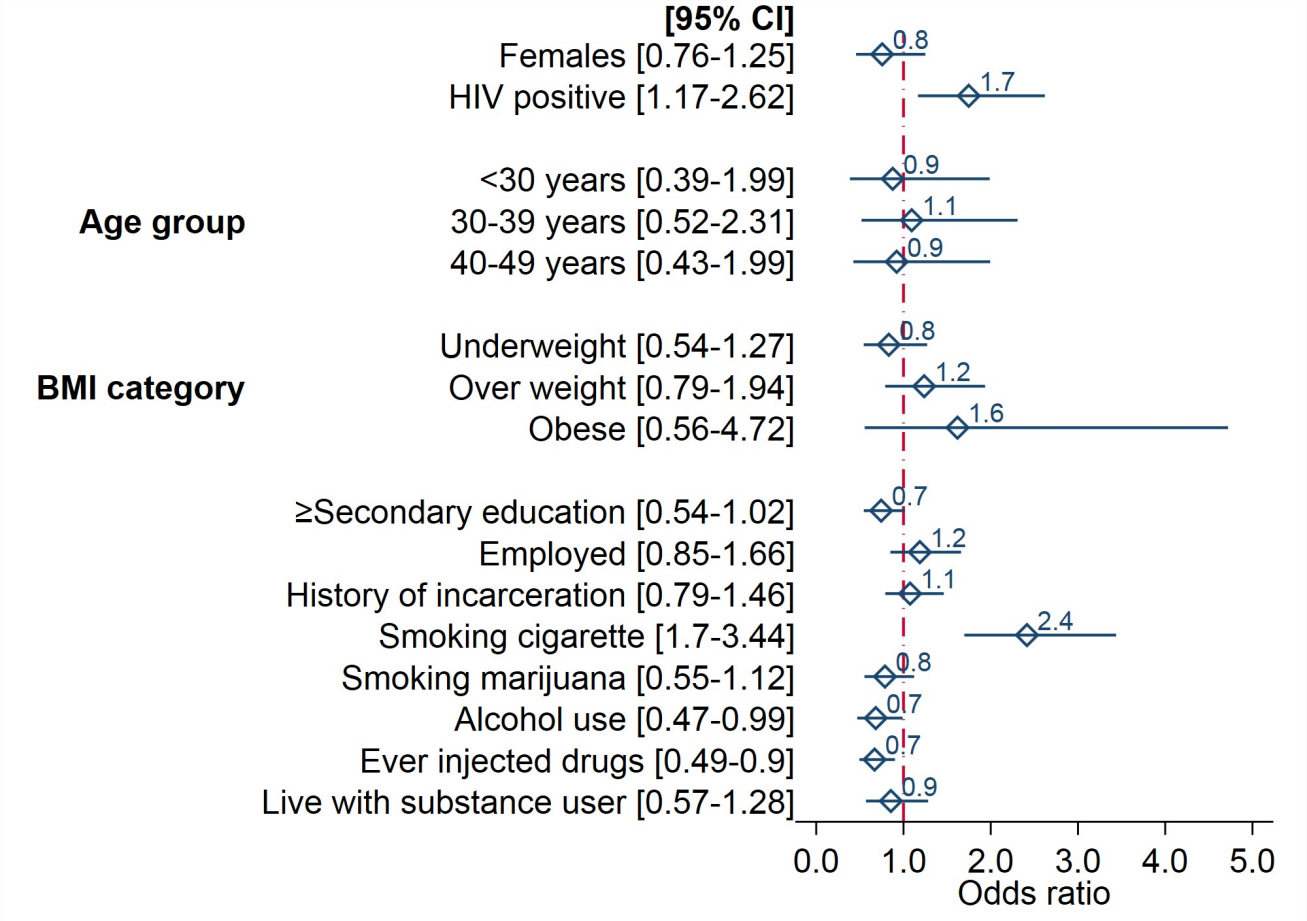
Risk factors of TB infection among people receiving medication-assisted treatment for substance use disorder in Dar es Salaam, Tanzania; adjusted logistic model (n = 823).

A total of 823/901 (91%) were analysed for TB infection after excluding prevalent and presumptive TB cases and those with unknown HIV status and missing TST results. Results for risk factors of TB infection among people receiving MAT for substance use disorder are shown in Fig 2. In adjusted logistic regression model adjusting for age, sex, HIV status, previous history of TB, BMI, education, occupation, ever injected drugs, alcohol use, smoking and incarceration or jail detention in the last 2 years, the risk of TB infection was higher in HIV positives compared to HIV negatives (aOR 1.75, 95% CI 1.17–2.62), increased in cigarette smokers compared to non-smokers (aOR 2.41, 95% CI 1.70–3.44) and reduced in PWID and alcohol users (aOR 0.67, 95% CI 0.49–0.90 and aOR 0.68, 95% CI 0.47–0.99 respectively).

### Incidence of TB disease

**Fig 3:**
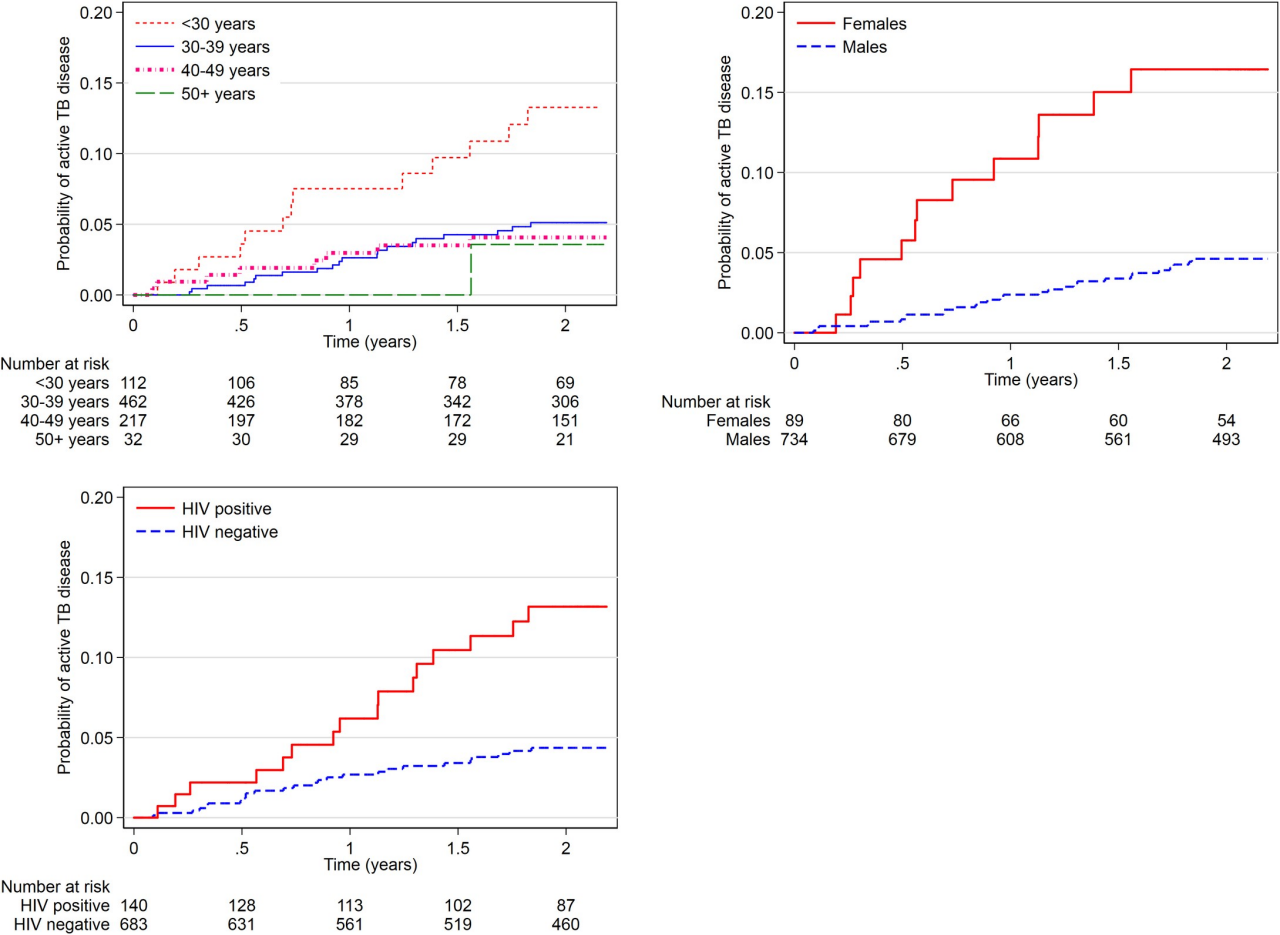
Factors associated with incident TB disease among people receiving medication-assisted treatment for substance use disorder in Dar es Salaam, Tanzania; Kaplan Meier plot (n = 823, incident TB = 42).

In total, 42/823 (5.1%) participants received TB treatment during the 22 months follow up period. Diagnosis of TB disease was made in 36/42 (86%) through a positive sputum sample, 3/42 (7%) by chest radiographs and 3/42 (7%) were clinically diagnosed. Distribution of TB disease cases in the 3 MAT clinics was 7/42 (17%), 27/42 (64%) and 8/42 (19%) at Muhimbili, Mwananyamala and Temeke MAT clinics respectively. The follow-up time was 1440 person-years with an overall incidence of TB disease of 2,925 patients per 100,000 person years (95% CI 2161.8–3958.3). The median follow-up time was 25 months. Table 2 presents the univariable and multivariable results of potential risk factors associated with incidence TB disease. As shown in Fig 3, in adjusted Cox model adjusting for age, sex, HIV status, previous history of TB, BMI, education, occupation, ever injected drugs, alcohol use, smoking and incarceration or jail detention in last 2 years. Females had 3 times increased risk of incident TB disease compared to males (aHR 3.17, 95% CI 1.47–6.83, p = 0.003). Patients aged 30–39 years (aHR 0.36, 95% CI 0.17–0.75, p = 0.006) and those aged between 40–49 years (aHR 0.30, 95% CI 0.12–0.76, p = 0.012) had a reduced risk of incident TB disease as compared to those aged less than 30 years. Being HIV positive doubled the risk of incident TB disease as compared to HIV negatives (aHR 2.07, 95% CI 1.02–4.21, p = 0.045).

**Table 2:** Risk factors of incident TB disease among people receiving medication-assisted treatment for substance use disorder in Dar es Salaam, Tanzania (n = 823, incident TB disease = 42).

Characteristics	Univariable	Multivariable
	HR (95% CI), P-value^a^	HR (95% CI), P-value^a^^,^^b^
Age categories, years		
<30	Reference	Reference
30–39	0.36 (0.18–0.73), 0.004	0.36 (0.17–0.75), 0006
40–49	0.30 (0.13–0.73), 0.008	0.30 (0.12–0.76), 0.012
50 and above	0.24 (0.03–1.84), 0.17	0.28 (0.04–2.25), 0.23
Sex		
Male	Reference	Reference
Female	4.02 (2.09–7.74), <0.001	3.17 (1.47–6.83), 0.003
HIV Status		
Negative	Reference	Reference
Positive	3.07 (1.65–5.72), <0.001	2.07(1.02–4.21), 0.045
Previous history of TB		
No	Reference	Reference
Yes	1.52 (0.73–3.18), 0.27	1.78 (0.80–3.96), 0.16
Body Mass Index (BMI) categories		
Underweight	1.79 (0.88–3.69), 0.11	1.60 (0.75–3.43), 0.23
Normal	Reference	Reference
Overweight	0.43 (0.10–1.79), 0.24	0.39 (0.093–1.66), 0.20
Obese	1.49 (0.20–10.99), 0.69	2.08 (0.27–16.06), 0.48
Education		
Primary or less	Reference	Reference
Secondary or more	0.95 (0.49–1.82), 0.87	0.92 (0.46–1.82), 0.80
Occupation		
Unemployed	Reference	Reference
Employed	1.19 (0.62–2.29), 0.60	1.62 (0.8–3.30), 0.18
Ever injected drugs		
No	Reference	Reference
Yes	0.93 (0.51–1.71), 0.82	1.05 (0.55–2.0), 0.89
Alcohol use		
No	Reference	Reference
Yes	0.81 (0.41–1.61), 0.55	0.72 (0.33–1.60), 0.43
Smoking cigarettes		
No	Reference	Reference
Yes	0.91 (0.48–1.73), 0.78	1.26 (0.58–2.77), 0.56
Smoking marijuana		
No	Reference	Reference
Yes	1.11 (0.57–2.13), 0.78	1.38 (0.64–2.99), 0.41
Incarceration or jail detention (last 2 years)		
No	Reference	Reference
Yes	1.30 (0.69–2.44), 0.42	1.59 (0.8–3.15), 0.19

^a^Hazard ratios, 95% CI and P-value obtained from Cox regression.

^b^Adjusted for age, sex, HIV status, previous history of TB, BMI, education, occupation, ever injected drugs, alcohol use, smoking and incarceration or jail detention in last 2 years.

## Discussion

This study generated important data on the high burden of TB infection and disease among clients receiving methadone in Dar es Salaam, Tanzania. First and most importantly, we could show that the incidence of TB disease among PWUD attending MAT clinics is at an exceptionally high level with 42 new TB disease cases among 823 clients within a 22 month time period, which is equivalent to an incidence rate of 2,925 patients per 100,000 person years with a 1440 person-years follow up time. This is twelve times the estimated national incidence of TB disease. Second, we established that the prevalence of TB disease among PWUD at 2.6% is slightly lower than the previously reported prevalence of 4% for people who exclusively use injectable drugs (PWID) [14]. Third, we demonstrated that the prevalence of TB infection in this population is considerably high as prevalence expected in the Tanzanian general population. Finally, we found a notably high HIV prevalence of 44% among women and 16% among men with an overall HIV prevalence of 19%. These results are very critical not only for TB programmes but also for TB/HIV disease control programmes in Tanzania, where effective strategies are critical to achieve WHO’s End TB goals by 2035.

To our knowledge this is the first study investigating the incidence of active TB disease among PWUD in Tanzania. This group is known to have an increased risk of TB, which can be explained by poverty and other social determinants, HIV infection and the physiologic effect of drugs on the immune system [8–10, 12, 13, 23]. The alarmingly high TB disease incidence rate is most likely an indicator of intense TB transmission among PWUD with the risk of spill over to health care workers and the general population. Sharing air space and inhaling droplet aerosols produced by a person with pulmonary TB are the key drivers of *Mycobacterium tuberculosis* (*M*.*tb*) infection and disease. PWUD often congregate in crowded places with poor ventilation, and these spaces have been identified as transmission hotspots [8, 24–26]. The accumulation of new cases in one of the three MAT facilities is even suggestive of an outbreak during our observation period. However, the discrepancy of disease burden between the sites remains subject for further investigation. More so, the highly mobile nature of PWUD further leads to fast spread of *M*.*tb* on the one hand, and multiple exposures to *M*.*tb* with the risk of reactivation due to increased mycobacterial load on the other hand [27]. In absence of molecular-epidemiologic data it remains to some extent speculative if new TB cases were mainly a result of re-infection through recent transmission or progression to active TB disease of an existing TB infection [28]. Yet, TST-positivity did not predict TB disease incidence, similar to other studies [29], suggesting that TB transmission might play the more important role. Previous studies among PWID suggested that incarceration and male gender are strong risk factors for TB disease [8, 23]. Interestingly, in our study the incidence of TB disease was not associated with history of incarceration or jail detention. This could be due to the fact that we looked at a two-year prior history of incarceration or jail detention considering the risk of developing TB disease is highest during the initial years following exposure.

The risk of incident TB disease was higher in females as compared to males. This is likely explained by the higher HIV prevalence among female participants in the study. Female PWUD are also more likely to be exposed to other TB risk factors such as smoking and alcohol use closing the male female TB disease gap. Furthermore, close interactions with multiple sex partners [30] not only adds to their risk of acquiring but also the onward transmission of TB and HIV to their partners and the community at large.

Prevalence of 2.6% TB disease and 54% TB infection is alarming, prompting deliberate initiatives for prevention and control of the epidemic. Although the prevalence rate of TB disease is slightly lower as previously reported from one of the three MAT clinics [14], this might be explained by inclusion of all people who use drugs regardless of the method of use in our study. Highly sensitive sputum culture was also not performed in our study, whereas Gupta et al. [14] reported 31% of symptomatic participants being diagnosed sputum culture-positive. Furthermore, a majority (96%; 134/140) of the HIV positives were registered into the HIV clinics and were on anti-retroviral therapy (ART), with studies showing that ART reduces the risk of developing TB disease among HIV positive individuals [31].

It is well established that after the highest peak of TB disease in infancy the disease risk declines and starts rising again during adolescence and adulthood [32–35]. Likewise, our study shows that TB disease among PWUDs follows a similar trend as those under 30 years of age had an increased risk of incident TB disease compared to those who were 30–49 years. While high rates of TB disease in infancy and the elderly are considered to be related to age-specific differences in the immune response, adolescent and adulthood TB disease is driven by increased exposure as well as progression of untreated LTBI [35].

Smoking cigarettes was also associated with an increased odds of TB infection, similar to a study that showed an increased risk of TB infection among smokers and ex-smokers compared to non-smokers [36]. Other studies have also shown that cigarette exposure, whether passive or active increases the risk of TB infection, reactivation TB, severity of cavitary TB disease and death [7, 37]. Cigarette smoke also prolongs the infectivity duration leading to prolonged transmission time [38].

A notably high HIV prevalence of 19% among PWUD was observed, which corresponds to 23% and 14% among PWID and Non-Injection Drug User’s, respectively. Our findings are slightly higher than the 15.5% reported for PWID in Tanzania by the UNAIDS however lower than other prevalence studies with estimates ranging between 34.8% and 51.1% for PWID in Tanzania [39, 40]. This could be explained by the overall reduced HIV prevalence in the country through measures such as the national test and treat policy, health education on HIV transmission and prevention, as well as daily directly observed intake of ART at the MAT clinics. Good adherence to ART leads to undetectable viral load and reduced HIV transmission. Similar to another study [41] we observed an overwhelmingly high HIV prevalence of 44% in females in spite of the low reported injecting practice, implying acquisition of HIV through factors other than injecting drugs.

Our findings have public health implications because PWUDs have largely driven TB disease epidemics in a number of countries [8]. After *Gupta et al*., described active TB disease prevalence of 4% among PWID in 2014, infection prevention and control measures for TB have been implemented at the MAT clinics. They include changing the layout of the clinic for better ventilation, integration of TB/HIV services including directly observed treatment (DOT) TB with a plan to cross train physicians and methadone clinic staff on management of TB/HIV and addiction [41]. Despite implementation measures, six years later, the burden of TB among PWUD on methadone is still unacceptably high. This situation calls for review and reinforcement of the TB control measures as well as new scientific approaches focused on this high-risk group. Targeted screening for active TB disease, e.g., using regular chest radiographies, preferably with computer aided detection (CAD), as a screening tool as well as contact tracing for active TB cases. The use of next generation Xpert MTB/RIF ultra assay should also be considered for TB disease diagnosis at the MAT clinics by the NTLP to enable early diagnosis to cut the on-going transmission and to allow timely and appropriate disease management [19, 42]. Furthermore, research using epidemiological, molecular genetic (‘DNA fingerprinting’), spatial analysis as well as mathematic modelling, e.g., based on carbon dioxide level, is needed to elucidate the transmission dynamics among PWUD [43, 44], a constantly growing population group in urban Tanzania.

Isoniazid preventive therapy (IPT) uptake was zero in our study despite the high HIV prevalence. Globally, studies have shown that TB preventive therapy reduces the risk of developing active TB by 33% and up to 64% in TST positive individuals [45]. Challenges such as drug side effects, long treatment duration, lack of reliable tests to rule out active TB, poor adherence to preventive treatment and hepatitis co-infection lead to low uptake on TB preventive therapy among clinicians and clients. Despite the fact that TB infection was not associated with incident TB disease, the vulnerability of acquiring TB disease remains high. The daily attendance to MAT clinics may provide the opportunity to give DOT for TB prevention among PWUDs while closely monitoring for adverse events and symptoms for TB disease.

One potential limitation of our study is that we used TST to test for TB infection. Prior BCG vaccination and non-tuberculous mycobacteria infection are known to interfere with TST, but not with interferon-gamma release assays (IGRAs) [46]. Nevertheless, the use of TST in our study is supported by WHO recommendations indicating, that due to non-superior sensitivity IGRAs should not replace TST in low- and middle-income countries for the diagnosis of LTBI in individuals living with HIV-infection [47].

Further limitations are related to way data were collected: i) self-reported data may have led to under ascertainment of some potential risk factors; ii) asymptomatic patients did not undergo investigations to rule out TB disease with studies showing detection of TB disease among asymptomatic clients [48]; iii) masking of TB symptoms with opiates cannot entirely be ruled out leading potentially to delayed or missed TB disease diagnosis; and iv) TB treatment was used as an indicator of TB disease, which might exclude untreated TB disease cases from the analysis.

## Conclusion

PWUD attending MAT clinics bear an extremely high burden of TB and HIV and are known to have driven TB epidemics in a number of countries. Our reported TB disease incidence is twelve times that of the general Tanzanian incidence, further emphasizing that this group should be prioritized for targeted TB screening, testing and treatment. Gender specific approaches should also be developed as female PWUDs are markedly more affected with HIV and TB disease than male PWUDs. TB control measures in the MAT clinics should also be intensified.
